# Analyzing the Role of Values and Ideals in the Development of Energy Systems: How Values, Their Idealizations, and Technologies Shape Political Decision-Making

**DOI:** 10.1007/s11948-024-00463-7

**Published:** 2024-02-29

**Authors:** Joost Alleblas

**Affiliations:** Department of Values, Technology and Innovation, Technical University Delft, Delft, The Netherlands

**Keywords:** Political ideals, Political discourse, Energy utopias, Technological feasibility, Political feasibility

## Abstract

This study examines an important aspect of energy history and policy: the intertwinement of energy technologies with ideals. Ideals play an important role in energy visions and innovation pathways. Aspirations to realize technical, social, and political ideals indicate a long-term commitment in the design of energy systems, distinguishable from commitment to other abstract goals, such as values. This study offers an analytical scheme that could help to conceptualize these differences and their impact on energy policy. In the proposed model, two spheres of interaction are highlighted: a material sphere in which values and technologies co-evolve, and an imaginary sphere in which ideals interact with idealized technologies. Furthermore, the relation between these two spheres can be understood in different ways. This study examines three cases that are illustrative of the different roles of ideals in the development of energy technologies and visions: (1) the evolution of safety in nuclear reactor design; (2) visions of atomic power in France; (3) the political idealization of a tidal power scheme in the Severn Estuary. Finally, the developed model implies more general insights for the development of sociotechnical systems. Amongst others, it shows why certain projects and technologies remain a political, but not a techno–economic option.

## Introduction

The cultural history of energy (systems) is rife with ideals and utopian visions that have become connected to emerging technologies for the generation, distribution, and storage of energy. Historians have shown how the electrification of America at the turn of the twentieth century was more than a technological transformation (e.g., Granovetter & McGuire, 1998; Hughes, 1993). This electrification was informed by diverse normative visions of new, idealized ways of living made possible by electricity (Nye, 1990).

Alternatively, changes in energy systems can also inspire nostalgic, idealized versions of the past. Limmer and Zumbrägel (2020) discuss how innovations in hydropower in Germany led to a romantic idealization of the ‘dying waterwheel’ as emblematic of pre-industrial means of energy use. Hanel and Hård (2015) argue that nostalgic sentiments regarding heavy-water reactors in both Sweden and former West Germany in the 1960s and 70s delayed the adoption of the more efficient light-water reactor through an appeal to an idealized ‘nuclear tradition’.

The idealization of past and future states of affairs, made possible by energy technologies, can take many forms. This study focuses on the idealization of *future* states of affairs related to the development of energy systems through the adoption and persistence of a certain type of moral goals: ideals. It shows that the pursuit of these ideals through technological innovation can have both a positive and negative impact on the development of energy systems.

In brief, the emergence of these ideals can be sketched as follows: First, the history of energy systems shows how emerging and existing elements of these systems create new, morally problematic situations. This means that actors, through their engagement with energy systems, encounter situations in which previous moral responses no longer work. Second, these actors might then start to create new norms, practices and values to cope with these situations. For instance, Mitchell (2011) shows how, over time, social justice and equality concerns emerged in the coal mines of northern France. More recently, the transition to renewable energy resources has been related to concerns for ecological sustainability and intergenerational justice raised by fossil fuel use (van de Poel & Taebi, 2022). Third, problematic situations might also lead to the adoption of new ideals. This happens when values come to be seen as absolute, universal, and uncompromisable, turning these values into often infeasible principles of moral conduct and engineering design.

Thus, morally problematic situations, encountered in the operation and development of energy systems, may lead to the adoption of new energy practices, new values and ideals. The latter two are here both understood as abstract moral goals, but ideals are distinguished from values in the sense that they are uncompromisable goals for which their feasibility is not a concern. Ideals may thus be seen as idealized values (see "Ideals and Values in Energy Systems" section). Often, around ideals what I call **utopian visions** are formed. These visions offer a blueprint for the ‘realization’ of a certain ideal, showing the sociotechnical configuration that allegedly realizes the ideal. In the case of energy, I argue, these visions often rely on (promising) technologies that seem to enable energy systems to become perfectly safe, perfectly just, perfectly secure. These technologies, then, become idealized themselves.

Sometimes, these utopian visions include the type of monumental projects that seemingly realize a perennial ideal in one stroke. The history of energy offers many examples of such utopian plans addressing morally problematic situations, but failing to materialize. Hermann Sörgel’s Atlantropa project (1932), for instance, envisaged a hydro-electric dam across the Strait of Gibraltar, solving Europe’s electricity needs while, at the same time, unifying Europe’s belligerent states (Gall, 1998, 2006). The Qattara depression hydropower project (approx. 1930) in the desert of Egypt proposed the inlet of seawater in a natural depression via a tunnel connecting to the Mediterranean Sea (or, alternatively, to the Nile). The project was again discussed as important for realizing Egypt’s 2030 plan for clean energy while also cultivating the area (Elsayed & Ismaeel, 2019). However, researchers have pointed out its negative environmental effects, as well as its costs compared to other clean electricity solutions—such doubts in fact go back to 1982 (Ibrahim, 1982).

Another such hydropower project is analysed in this study. I discuss a large tidal power project proposed in the Severn Estuary between Wales and South–West England. A tidal barrage for energy generation in this estuary has been discussed since the 1880s, but has never materialized. In this tidal power project, a persistent political ideal allegedly comes within reach through a single project. However, once the feasibility of the project is considered from other perspectives, such as environmental effects, costs, and social consequences (e.g., displacements, job losses, loss of cultural heritage) doubts ensue. Consequently, the project loses much of its appeal though not necessarily its political support (Gall, 2006).

Such cases show that ideals have played an important role in the history of energy, helping to understand why, for instance, technological projects do not lose their political attraction, regardless of their techno–economic feasibility. However, despite a wealth in historical examples, the relation between ideals, values, and technologies needs more analysis. This study provides such an analysis. It proposes a new model to understand how ideals, values, and technologies interrelate in the development of energy systems. This model helps in understanding the complex interaction between political discourse, visions, and constraints as they develop over time. As illustrations of specific aspects of this model, this study presents three cases: 1. The evolution of safety in engineering design, with a focus on design for safety in nuclear reactors. 2. The role of ideals in the development of nuclear technologies in France; showing how utopian visions of a future France impacted design decisions. 3. The aforementioned analysis of the Severn Barrage tidal power scheme.

Through these examples, this study provides more general insights into the role of ideals in the development of sociotechnical systems. Amongst others, it shows how (once) promising technological projects could become entangled with certain ideals in political discourse, making it difficult to relinquish these projects. In this entanglement, it can become difficult to distinguish socio–political ends from technological means. The Severn Barrage tidal power scheme is presented as an example of this entanglement.

"Ideals and Values in Energy Systems", "Ideals and Energy Visions" and "Ideals and Engineering Design" sections elaborate on the role of values and ideals, and the distinction between them, in engineering design and the development of energy systems. "Inherent safety in reactor design" and "The role of ideals in visions of energy system development" sections focus on ideals in the development of nuclear technologies and further develop the proposed model. "The Severn Barrage" section discusses the Severn Barrage tidal power scheme. "Discussion" and "Conclusion" sections provide the discussion and conclusion.

## Ideals and Values in Energy Systems

Over the last decades, a wide range of approaches has been proposed to capture the way moral considerations influence the design of technologies, the choice of certain technological and scientific projects, and the selection of innovation pathways. The Value Sensitive Design [VSD] literature, for instance, investigates how salient ethical values can be embedded in technological artefacts (Friedman, 1996; van de Poel, 2009). Research into technological mediation traces how technology mediates the relation between humans and their lifeworld, leading to new moral considerations, and eliminating others (Verbeek, 2006). Furthermore, literature in Science and Technology Studies has used the notion of the 'sociotechnical imaginary' to explore the role of collective conceptions of the good life in the development of sociotechnical systems (Jasanoff & Kim, 2009, 2013), systems in which humans and technical elements conduct goal-oriented behaviour, thus providing a social good (Walker et al., 2008). Research into these sociotechnical imaginaries offers an analysis of how anticipatory discussions of possible and desirable futures determine specific policy decisions in the present (Stilgoe et al., 2020).

Despite the interest in idealized technologies and technological utopias, or ‘technotopias’, ideals can be difficult to conceptualize and distinguish from other abstract goals, such as values (Coady, 2008). Following Van de Poel’s (2009) account of values in engineering design, this study takes ideals to refer to stable goals that transcend specific situations as well (see Table 1). Indeed, stability and abstractness are shared characteristics of values and ideals. Furthermore, values might start to function as ideals over time, and vice versa. Likewise, what is considered ‘utopian’ might differ over time and place. However, ideals are uncompromisable and often unfeasible, whereas values are not (Rescher, 1987). This has implications for how values and ideals function in engineering design and innovation.

**Table 1:** Definition of terms

Value	Stable, abstract goal transcending specific situations, while at the same time specifying the variety of ‘goodness’ of certain states-of-affairs, such as a sustainable home or a safe car
Ideal	Stable and uncompromisable abstract goal for which its feasibility is not a concern; often appearing in the form of a (universal) principle for action, or a perfect state-of-affairs, such as an ideal body, or an ideal society
Vision	Blueprint for the future realization of a value or ideal, showing which institutions to create, which technologies to embrace
Utopia	Blueprint of a future society in which an ideal or set of ideals is realized: a perfectly just/free/autonomous/healthy society; often based on the embrace of a specific set of technologies
Energy Utopia	Blueprint of a future state of the energy system in which (a set of) energy ideals are fully met in the design of material (technological) and immaterial (institutional) aspects of the energy system

Notwithstanding the vagueness of the boundary between ideals and values, in the case of engineering design, we are able to make a distinction between them: because of their uncompromisable character, ideals do not 'materialize' in technological artefacts in the manner that values can. Ideals are by definition not completely feasible and therefore can never be fully realized in a certain design. This means that ideals are instructive for innovation trajectories, but cannot become embedded in specific technological artefacts and systems. Because of their unfeasibility, engineers do not have the possibility of translating ideals into (realistic) design requirements for specific technologies, like they do for values—as the VSD-literature proposes (van de Poel, 2013). Ideals will never be fully realized because of the concessions engineers and designers need to make while designing for certain salient values. Instead, because ideals motivate and orient actors in their decisions, we can find them in visions of the future, especially as these visions are often 'encoded and decoded as utopias and dystopias' (Berkhout, 2006). Striving to realize these ideals would then have an impact on the kind of decisions made concerning the design of energy systems, as the coming sections makes clear.

## Ideals and Energy Visions

Ideals have been extensively discussed in political philosophy. In these discussions, certain social ideals, such as ideal justice, motivate institutional change (e.g., Estlund, 2019; Gaus, 2016; Rawls, 1971). Questions of feasibility play an important role in these debates. For instance, Rawls (1971) calls his vision of a just society, achieved through institutional reform, a ‘realistic utopia’, emphasizing the tension between political hope and realistic ambition.

In discussions of visions for the development of sociotechnical systems, existing literature seems to refrain from *explicitly* addressing the impact and function of ideals, as well as the question of feasibility. It is unclear, for instance, to what extent Jasanoff and Kim's (2009, 2013) sociotechnical imaginary has ideal or utopian aspects. The good life that a society collectively aspires to—as a desirable future that shapes and supports national technoscientific trajectories—might very well be imaginable but hardly feasible. Jasanoff and Kim seem to give no answer to the question what happens when the pursuit of collective ideals is in fact hampered by the technoscientific projects that are collectively chosen on the basis of these ideals. Furthermore, these imaginaries might differ in degree of their feasibility, and might differ in the degree to which the abstract goals around which these imaginaries are formed, are compromisable. A certain vision of social order might, over time, prove unfeasible. One could then either abandon it, and adopt alternative imaginaries, or continue pursuing it.

**Fig. 1:**
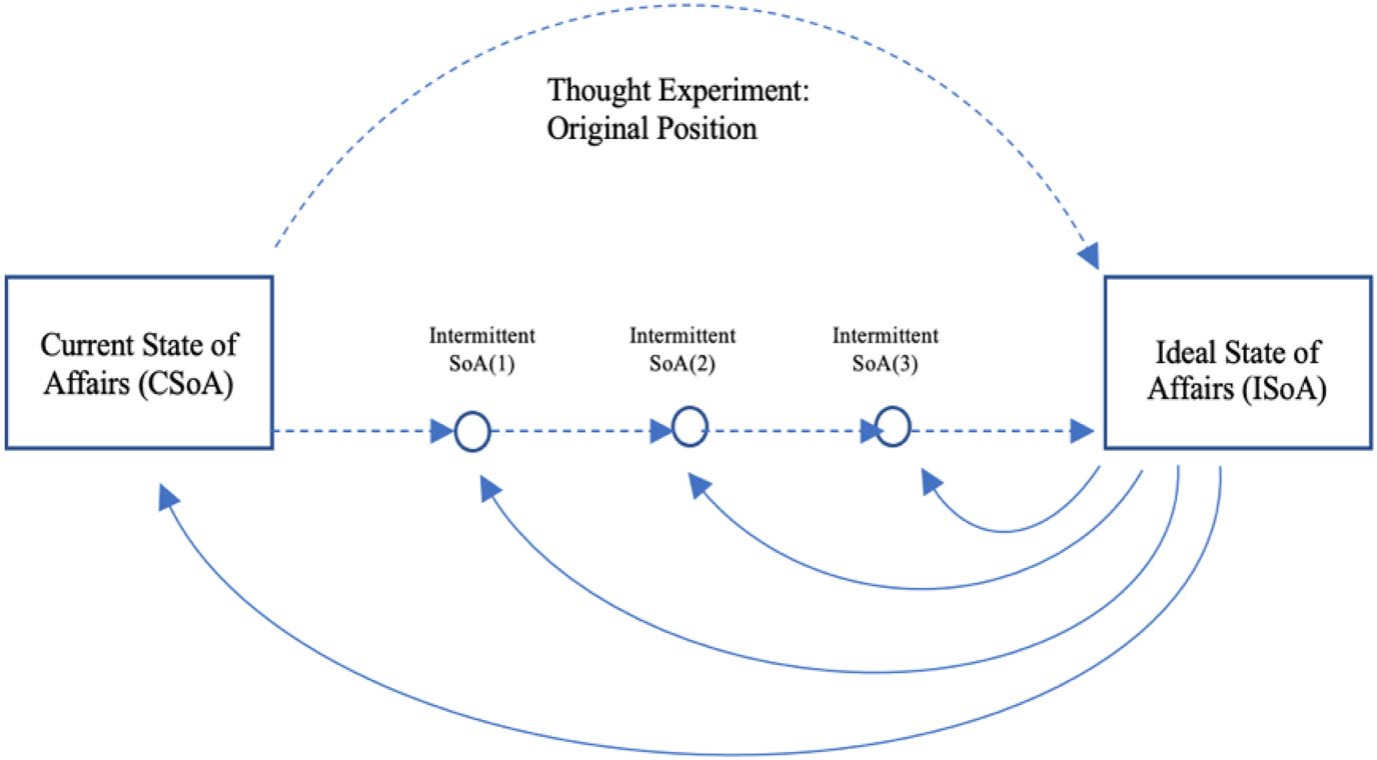
The creation and function of ideal justice

One might also intentionally develop an unrealistic collective vision of a desirable future (an abstract ‘moonshot’), as a long-term navigational tool to inform decision-making regarding social and technological issues.^1^ Such, some authors claim, is the role of ideal justice (Estlund, 2019; Rawls, 1971).^2^ Figure 1 shows this navigational function. The Ideal State of Affairs [ISoA] is needed to decide upon the right course of action in Intermittent States of Affairs [Intermittent SoA]. A thought experiment is used to arrive at a set of shared principles of justice that form the basis for an ideal institutional configuration in the ISoA. This ISoA, as utopian vision, then informs decision-making at critical junctions on the road to a fully just society (see Fig. 1). At each junction, therefore, a teleological evaluation is made in which the absolute end-goal determines the rightness of the decision—and the actions proceeding from it.

We might similarly perceive the role of ideals in other processes of decision-making, e.g., political decisions concerning the future of an energy system or design decisions made by engineers. In these cases, the formulated abstract ambition functions as a long-term beacon, deciding the choice of technologies, materials, etc. The ideal can be a moral principle, one that actors know they cannot always comply with in their actions (such as principles of ideal justice, ideal safety, etc.), or a utopian vision of a future state of affairs in which these limitations have disappeared. In these cases, the role of ideals in engineering design is teleological, rather than evolutionary and adaptive—as I see the role of values. These different roles are further developed in the next sections.

## Ideals and Engineering Design

Moral ideals often come in the form of universal principles for action. They are, in a normative sense, ‘conceptions of genuine perfection’ (Brownlee, 2010, p. 434) that concern the performance of ourselves as agents, of our institutions, or of our technologies.^3^ Holding on to such guiding principles (e.g., to never lie, never hurt an animal, to build a completely sustainable economy or a completely just society) might come with considerable effort and costs, while on a personal level, attaining perfection might not always be desirable (Wolf, 1982). Still, on a societal level, benefits could result from adopting these principles, even if they are often impossible, impractical, and unachievable (Rescher, 1987). First, benefits result from the presumed navigational function of ideals. For some authors, ideals facilitate the comparison of possibilities in the present with regards to their proximity to a formulated ideal.^4^ Second, according to most definitions ideals aim for the absolute (Rescher, 1987; Rawls, 1971; Nozick, 2013). This means an ideal, as end goal, surpasses the merely feasible, understood as what actors can achieve given historical, local, physical, biological and social or psychological constraints. An ideal, therefore, challenges actors to perform to the limit of their abilities (e.g., Rescher, 1987).

Despite their unfeasibility, societies strive to ‘realize’, or at least to approach, moral ideals. This means the ideal is collectively recognized as desirable, while its full realization requires a level of (human) perfection that is highly unlikely to be achieved. There seem to be two reasons why actors may not be able to comply with an ideal as moral principle in their actions. On the one hand, there could be external obstacles that inhibit them from following the moral principle. This means they perceive themselves as unable to act in accordance with the principle. On the other hand, we can think of internal obstacles that cause social agents to make an exception in certain situations. In this case they perceive themselves as unable to will the act in accordance with the principle.^5^ Both forms of inhibition are no longer present in a utopian vision, all agents are assumed to be able and willing. At that moment the principle truly becomes a universal moral law, and exceptions no longer exist.

**Fig. 2:**
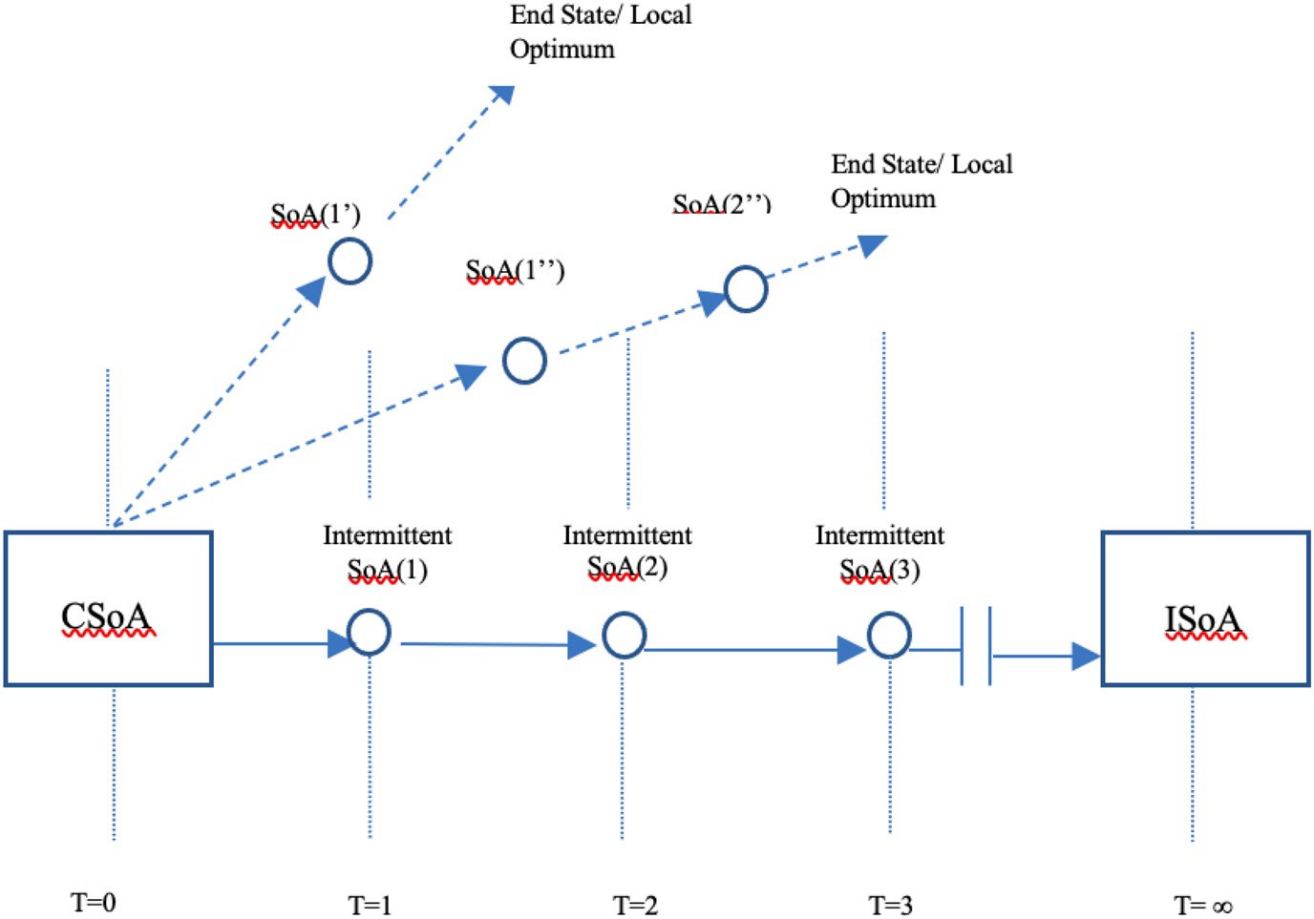
The navigational function of ideals, showing us the way from a current state of affairs (CSoA) towards absolute (ISoA), instead of local optima (dashed lines, indicating innovation trajectories without ISoA’s)

In the meantime, before this realization, visions help in navigating real-world situations of imperfection. As Gaus (2016) argues, holding on to ideals would mean striving for an absolute optimum, instead of a local one.^6^ A local optimum occurs when goals are realized in conjunction with other, and often incompatible, goals. An absolute optimum occurs when such a goal is an ideal, and no longer in competition with other goals. This ideal helps societies distinguish between local and absolute optima while they navigate political, technological, and technopolitical landscapes and futures.^7^ At the same time, as a navigational tool, it makes sense to require from an ideal an accompanying, and detailed, itinerary how to achieve it (see Fig. 2).

Taking the presumed comparative function of ideals into account for innovation trajectories, another important difference emerges between values and ideals in engineering design. Approaches such as design for values offer an evolutionary, adaptive perspective on design, in which value conflicts (e.g., between security and privacy, sustainability and safety) are seen as issues that can be resolved, amongst other strategies, through innovation (van de Poel, 2009, 2015; van den Hoven et al., 2012). An absolute end-goal is missing. Innovation processes might stop when an acceptable solution to a value conflict is achieved.

**Fig. 3:**
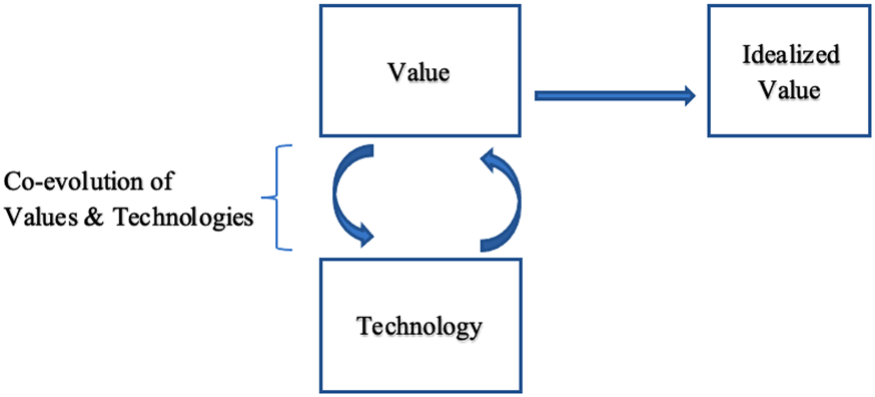
Distinguishing values from ideals in technology design

Holding on to ideals has consequences for the way we innovate in engineering design. Again, I use Gaus' (2016) distinction between local and absolute optima to make this clear. In technological innovation, I argue that a local optimum occurs through incremental change to realize a certain salient value, such as safety or privacy, taking into account material and other constraints (see Fig. 3, left-hand side).

Designing to incorporate a certain value, means to maximize it given the circumstances. We can also imagine achieving in design a minimal, required ‘threshold’ level for a certain value, as outlined in the VSD-literature.^8^ This idea of design we could understand as an evolutionary process of adaptation to new circumstances, such as new societal demands or higher threshold levels. As ideal, however, we envision an absolute realization of a certain goal, an absolute optimum, without having to compromise the goal given the demands of other values and goals with which it might be in conflict (see Fig. 3). This process is teleological, meaning that the often unfeasible end-point is clear from the start.

**Fig. 4:**
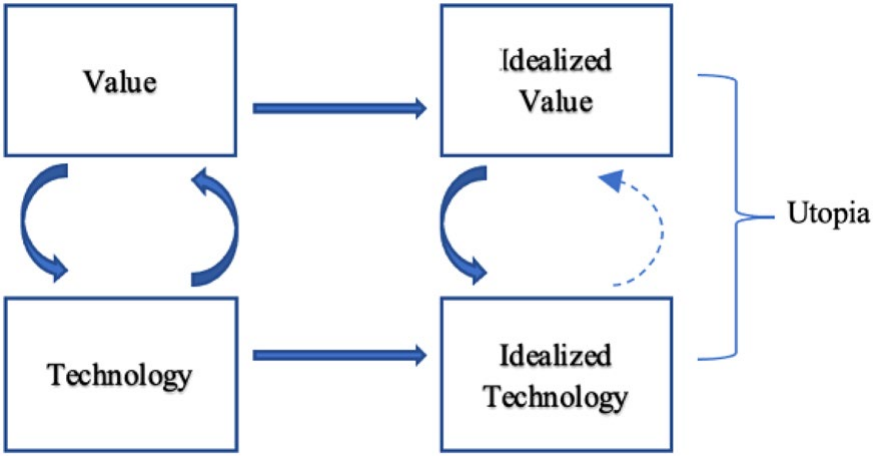
Utopian configuration in technology design

A technology that is imagined to fully realize an ideal, we could call 'an idealized' technology. Such a technology is therefore envisioned, but never realized. The result of the interaction between an ideal and an idealized technology, I refer to as an (technical) utopia,^9^ showing in what (sociotechnical) configuration a certain ideal, or set of ideals, will be achieved. This rudimentary distinction is shown in Fig. 4. Furthermore, the dashed line in Fig. 4 indicates a possible influence of the idealized technology on the idealized value. I will return to this possibility in "Discussion" and "Conclusion" sections.

## Inherent Safety in Reactor Design

As an illustration of Figs. 3 and 4, I provide an analysis of the evolution of safety in the design of nuclear reactors. Although the idealization of safety in reactor design is contested, this section argues that, at least from a political and public perspective, safety started to function as an ideal in discussions of nuclear technologies in the 1990s. This analysis, furthermore, highlights that thinking in an idealistic vein had (radical) implications for reactor design. As an ideal, inherent safety had a clear political and public function, attesting to an absolute, political commitment to this ideal. However, it is less evident that the ideal continues to be pursued through reactor design.

At first safety functioned as a value amongst other values in reactor design, such as sustainability, security, and economic viability (see Fig. 3, left-hand side). The first safety approaches entailed what was later called ‘active safety’, the value of safety was supported by active involvement of an operator monitoring processes to impede a reactor core meltdown (Taebi & Kloosterman, 2015). Hazards needed to be controlled through this active involvement. Increasing active safety meant adding more monitoring systems, further complicating the role of the operator of nuclear power plants. Furthermore, reactor safety was an incremental objective: an aspiration to achieve a local optimum (see Fig. 2), given the then existent particulars of the Light Water Reactor-design, and given the restraints the realization of conflicting values put on reactor safety (Taebi & Kloosterman, 2015).

This situation, however, changed drastically with the occurrence of the first nuclear accidents, most notably the one in Three Mile Island (1979) and Chernobyl (1986). These accidents made safety a public priority in Generation II reactors (1965–1996). The rationale was that existing Generation II reactors needed to be made much safer. Furthermore, with the anticipated growth of nuclear energy—referred to as the ‘nuclear renaissance’—the occurrence of nuclear accidents and incidents was likely to rise too. In the 1980s, estimations of the number of reactors in the 1990s exceeded 5000, which was a tenfold of the operational reactors at that moment (Taebi & Kloosterman, 2015).^10^ This meant acceptable levels of safety as stipulated in public policy back then (1 serious nuclear accident per 10.000 reactor years, or years of reactor operation), prior to the 1980s, were no longer deemed sufficient: with ca. 500 reactors globally, that meant about 1 accident every 20 years. With the possibility of 5000 reactors in full operation, the likelihood of an accident would become about ten times higher; this meant the possibility of a serious accident every two years. Higher levels of safety were, however, difficult to achieve through the existing safety regime: active safety. Adding even more complex systems, more back-up generators, redundant pumps etc. would on the one hand increase safety but the complexity could also contribute to decreasing safety. A (local) optimum had been achieved that proved to be unacceptable. Existing safety regimes needed to be reconsidered.

Passive safety emerged in the 1990s as a new and complementary understanding of safety in reactor design, because of the aforementioned concerns with active safety. This new understanding meant designed safety features of reactors often became based on natural forces, such as gravity (INSAG-3; INSAG-5, 1992; Juhn et al., 2000). The use of these natural forces meant that safety was less dependent on active intervention by system operators or by pumps that required external power (Juhn et al., 2000). Passive safety features also reduced complexity, resulting in considerably safer reactors, reducing the probability of an accident. This probability was now estimated as lying in between 100,000 and a million reactor years, depending on the type and magnitude of the accident (IAEA, 1992). Both active and passive safety, however, continued to be considered in relation to other values, such as output reliability, security, economic viability, and ease of maintenance (Juhn et al., 2000; INSAG-5). Furthermore, improvements in safety were seen as ‘evolutionary’ (INSAG-5), or adaptive, rather than teleological, as would be the case with the pursuit of safety as an uncompromisable ideal. INSAG-5 attests to the limits of this evolutionary approach, stating:

There seems to be a limit to the benefits to be gained from evolutionary improvement of current designs. Three main factors set the basis for this limitation. These are: human factors in operation, the complexity of plants and limits on the benefit from confinement systems. (INSAG-5, p.50)

Inherent safety emerged from a new safety philosophy, a new way of thinking about safety. It is often conceptualized as an ideal (Fig. 3, right-hand side). In these articulations, inherent safety concerns the elimination of all inherent hazards instead of controlling them (IAEA, 1991; Kletz, 1978; Kletz & Amyotte, 2019).^11^ Instead of accepting that hazards are inevitable, and seeking solutions to reduce their probability or to mitigate their negative effects, the hazard should be completely removed. This elimination could only be ‘achieved’ through substantial changes in the reactor design; it often meant that a design from scratch needed to be proposed with inherent safety as the leading principle (Taebi & Kloosterman, 2015).

However, different interpretations of inherent safety were developed over time. In some of these accounts inherent safety functioned as a value, in others as an ideal.^12^ For instance, Weinberg (1985) does not equate inherent safety with absolute safety or a fail-safe design, but with passive safety. Even inherently safe reactors may suffer from accidents, in the range of once every 10^9^ reactor years (Core Melt Probability) (Weinberg, 1985; Spiewak & Weinberg, 1985; van de Poel, 1998). Despite functioning as a guiding principle for reactor design (van de Poel, 2003), the principle of inherent safety thus conceived is not an ideal. The IAEA, on the contrary, defined inherent safety as ‘equivalent to absolute safety; i.e., an inherent safety characteristic is not subject to failure of any kind. Stated another way, an inherent safety feature represents conclusive, or deterministic safety, not probabilistic safety’ (1991, p.10). But, for the IAEA, only components of reactors can be inherently safe, not reactors themselves, thereby undermining the aim of a new design approach for the reactor as a whole (van de Poel, 1998). To the public, furthermore, proponents of nuclear energy presented inherent safety as an ideal pursued in reactor design, especially in the 1990s (Barkenbus, 1988; Mårtensson, 1992; van de Poel, 1998). The inherently safe reactor was proposed as a technological solution to a political problem (Barkenbus, 1988). Researchers have focused on the public acceptability of nuclear risks (Barkenbus, 1988; Mårtensson, 1992; Adamov et al., 2015). In these accounts, inherent safety concerns the elimination of all socially unacceptable and therefore ‘important’ hazards.

Design for inherent safety was effective in redirecting technological development and changing the technological regime in reactor design. However, while an inherently safe reactor can be designed that cannot melt down, it is not inherently safe against other hazards and risks such as the risk of large scale radiation leakage. As public ideal, inherent safety cannot be achieved, since it is impossible to remove all hazards through nuclear reactor design. For that reason, Kletz and Amyotte (2019) instead discuss ‘inherently safer designs’, in which the 4 principles of inherent safety for chemical plants—intensification, substitution, moderation, and simplification—have been applied to *minimize* known hazards.^13^ Furthermore, not all accidents can always be anticipated, leading to the occurrence of ‘normal accidents’ as Charles Perrow (1999) calls them.

The extent to which inherent safety continues to function as a guiding ideal in the design of nuclear reactors, while having fundamentally altered the conceptualization of safety in this field, is therefore contested. While certainly presented as an ideal in public discussions of nuclear energy—as uncompromisable, absolute, and guiding long-term innovation trajectories—especially in the 1990s, it is unclear how and to what extent engineers continue to embrace inherent safety as an engineering ideal.

## The Role of Ideals in Visions of Energy System Development

The previous section has shown how the adoption of an ideal can lead to a radical rethinking of (reactor) design. On a larger scale, ideals can also impact the design and development strategies of energy systems. These strategies often rely on visions to make them socially and politically viable (Trutnevyte, 2014; Van der Helm, 2009; Ziegler, 1991). As they define some future state of the energy system, these energy visions can become utopian. Berkhout (2006) argues that visions are characterized by expressed objectives, suggested technologies, and are representative of a social order. Once the expressed objectives and required technological performance move further away from what is currently deemed possible, the vision starts to become utopian. In the case of far-reaching visions for energy systems, we can speak of energy utopias.

A clear example of such a utopian vision, we find in France, in the aftermath of WW2. This vision concerned the development of a nuclear energy system. The state agency that led the nuclear research program, the Commissariat à l’Énergie Atomique [CEA], created in 1945, envisioned a nation that would be completely sovereign, dominant in the geopolitical order, and self-sufficient in terms of energy generation, thus aligning energy policy with foreign policy (Hecht & Callon, 2009). Nuclear technologies (for both civilian and military purposes) were deemed quintessential for the realization of this utopian vision (see Fig. 4). This is what we could call an 'energy utopia’, since it idealizes a future state of affairs to be brought about by radical changes in the energy system, through idealized new technologies, and the goals that are achieved through it. As Hecht and Callon (2009) sees it, ‘They [the technologists] agreed on the ideal of a technologically radiant France, but they did not necessarily agree on the best route toward that ideal’ (p. 53).

The CEA’s vision of a radiant, nuclear France, however, did form a blueprint of a future society based on a radical redesign of its energy system. The blueprint was meant to convince French citizens of the necessity of nuclear energy and motivate short-term sacrifices in exchange for long-term gains (Hecht & Callon, 2009). Furthermore, in a political sense, the utopian vision led to specific technological choices in which three political and uncompromisable ideals (sovereignty, Frenchness,^14^ and technological prowess) consistently trumped values such as efficiency, and economic viability (Hecht, 1996; Hecht & Callon, 2009; Scheinman, 2015). At least, in the design decisions of the CEA, the state agency that dominated decision-making concerning the development of nuclear technologies until the late 1950s (Hecht & Callon, 2009).^15^

The decisions through which this utopian vision of nuclear proliferation was pursued include: the choice to build homemade reactors (gas cooled, using natural uranium) instead of importing North-American technologies, that ran on enriched uranium, which would have to be imported as well; the choice to have a closed fuel cycle that would allow for hosting facilities to produce weapons-grade plutonium; the construction of a costly loading system that wouldn’t require a shutdown of the reactor during loading and unloading enabling a fast extraction of plutonium; the decision not to optimize reactor design for goals such as electricity production or cost-effectiveness (Hecht & Callon, 2009).

These decisions made in the early stages of the French nuclear program show the pursuit of a utopian vision through design decisions of nuclear technologies. It is beyond the scope of this paper to analyse to what extent these ideals have been followed in the design of nuclear technologies, policies, and strategies in France while it was confronted with geopolitical events, nuclear disasters, and political conflict (see, for instance, Scheinman, 2015; Pelopidas, 2012, 2019; Jurgensen & Mongin, 2018). That is, this paper cannot give a detailed account of the abandonment of the utopian vision of the CEA.

However, some observations can be made. First, the return of De Gaulle as head of state, in 1958, meant that the CEA lost some of its independence. This meant that ‘conducting nuclear technopolitics now involved more than embedding pre-existing political goals into technological artifacts’ (Hecht & Callon, 2009, p.91). Second, the competing vision and corresponding reactor design of the Électricité de France [EDF] won ‘the war of the systems’ (Hecht & Callon, 2009). This design was focused on a wider range of goals, related to the industrial and commercial application of nuclear technologies. In other words, the utopian vision of the CEA was replaced with the more pragmatic vision of the EDF (Scheinman, 2015). Third, national sovereignty over time became a problematic ideal in the case of nuclear energy production, that required international agreements and international control (Taebi & Mayer, 2017). Fourth, recent struggles in Mali and Niger have shown that uranium isn't a conflict-free resource (Filippov, 2015; Keenan, 2008), and the ideal of energy sovereignty is liable to geopolitical changes and post-colonial struggles.

Therefore, despite the continued possibility of an unconditional pursuit of the aforementioned ideals from the perspective of engineering design, a design which would continue the vision of the CEA, politically this pursuit was no longer desirable. As absolute goals, these ideals became politically compromised, and started to function as values instead. Concessions therefore became necessary, value trade-offs occurred.

## The Severn Barrage

The previous two sections have focused on the productive force of ideals in engineering design and the development of energy systems. In contrast to the last section, that focused on the political abandonment of certain ideals despite their successful guidance in engineering design, this section shows an opposite process: the political pursuit of ideals through a politically idealized technological project, in which these ideals have no productive impact on engineering design.

Wave and tidal power have gained considerable interest in the UK as a stable renewable energy source. However, hardly any projects make it beyond the pilot stage. One project in particular stands out in terms of political commitment, research funds, and academic discussion: the Severn Barrage, a proposed tidal power barrier in the Severn Estuary, between Wales and the South-West of England. Interest in this barrage started in the 1880s already, but really took off in the middle of the 1970s, after the first Oil Shock. Since the 1980s, at least six feasibility studies have investigated electricity output, costs and a wide range of possible effects of this barrage, as well as its most favourable location in the Severn. Despite this continued political interest, the barrage was never realized.

Political procrastination in the UK regarding large infrastructure projects has gathered considerable academic attention. We can, therefore, give several possible explanations for this persistent political focus on the large tidal power scheme in the Severn, despite its apparent techno–economic infeasibility. First, some authors (e.g., Lijphart, 2012; Watson, 1992) have stressed the adversarial character of the Westminster, majoritarian model of government as a possible source. This model leads to competition and conflict. It is a ‘free-for-all pluralism’ (Lijphart, 2012), that doesn’t motivate coordination and cooperation. Second, Keay (2016) has suggested the UK is stuck in ideological limbo, as far as its energy policies are concerned. Third, an escalation of commitment might explain the persistence of this paper project. The political fixation on a project could occur because politicians are unable to accept the failure of a project, because they are too invested in it (Maxwell et al., 1997). It is possible that politicians have connected their reputation to this project, and perceive abandonment of the project as ‘ego-threat’ (Zhang & Baumeister, 2006).

Without denying the relevance of the aforementioned factors, I propose another perspective for the persistence of the Severn Barrage, as part of a far-reaching vison, an energy utopia. While no final decision on the barrage was made in the period of 1981–2014, ‘tidal power’ and the ‘Severn Barrage’ were mentioned approximately 2000 times (Table 2), in 378 separate debates in the House of Commons and House of Lords (hansard.parliament.uk).^16^ This continued political attention led to six consecutive feasibility studies that were unable to form final conclusions. Nor did consecutive UK governments draw them. The project was not abandoned, despite consistent findings that tidal power in the Severn, in its ‘current’ form, would not be able to compete with nuclear or wind energy in terms of costs per kWh (DECC, 2009a, 2009b; DBER, 2008; SDC, 2007). Despite an apparent technological infeasibility, political support continued. This support was partly grounded in the conclusions of each feasibility study, which repeatedly stated that changing external circumstances and innovation efforts might, one day, tilt the balance in favour of the Barrage.

**Table 2:** Word count Hansard archive

Term	Wordcount
Severn	1078
Barrage	430
Severn barrage	1445
Tidal	1731
Tidal power	606

Concluding, the interpretation this section proposes of the failure to make a final political decision concerning the Severn Barrage is the persistence of tidal power as part of an utopian energy vision connected to a range of ideals which were upheld in some corners of British parliament. While these ideals were unrealistic and unfeasible, they could persist as ideals because a lack of realization is often not an argument against holding certain ideals, as "Ideals and Values in Energy Systems" and "Ideals and Energy Visions" sections have made clear.

In a nutshell, these ideals led to aspirations and commitments regarding the Severn Barrage that the project was unable to fulfil and politicians were unable to discard. In this perspective, furthermore, the Severn Barrage itself seemed to serve as the blueprint for the realization of these ideals, as a *necessary* milestone. In this reading, abandonment of the Barrage, would also mean an abandonment of cherished political ideals, as means (technologies) and ends (ideals) became confused.

## Discussion

**Fig. 5:**
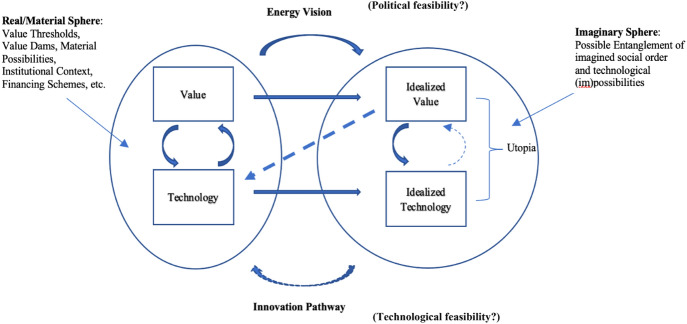
Innovation cycle in energy system development and/or transition

The three case studies indicate that the role of ideals in energy policy is multifarious. Ideals can have both a positive and negative impact on the development of energy systems. As a result of the three cases discussed, Fig. 5 presents 2 spheres of interaction: an imaginary sphere of energy policy, and the material sphere of engineering design. This figure represents what a (energy) vision does, in which certain salient values—in the current, engineering context still compromised by other goals and restraints—become idealized. Unfeasible standards and principles infuse future visions and become part of a utopia once they get linked to specific technological arrangements. These technologies can be already existent, or only imagined (such as certain geo-engineering technologies). In either case, these technologies are idealized as well; that is, functioning in full service of utopia, ‘realizing’ the ideals.^17^ Finally, this utopian blueprint of a future society can lead to the creation of a specific innovation trajectory (a pathway) that specifies how a technology can become what it should be.^18^

This updated model allows us to visualize a mechanism: A value could become more prominent over time (e.g., safety in reactor design, sustainability in energy systems, national energy sovereignty) and starts to be conceptualized as an ideal which affects idealizations of certain technologies (nuclear technologies, hydropower projects), or lead to a radical rethinking of technological regimes, such as in reactor design (dotted straight arrow). Secondly, while a vision formed around ideals and idealized technologies might affect the actual design of these technologies via an innovation pathway, the full realization of this vision remains technologically unfeasible. How actors react to this unfeasibility, determines whether or not the vision is abandoned. This reaction is possibly informed by the extent to which means and ends have become intertwined in utopian visions. If certain technologies and projects (means) are deemed necessary for the realization of one or more societal ideals (ends), the technology might replace the ideal as navigational beacon for innovation.

Two questions, furthermore, emerge. We can ask if the prioritization of a value, its idealization and henceforth uncompromisable character, is politically feasible. In this case, the question is whether we can ‘onboard’ all relevant stakeholders to accept the absolute, uncompromisable character of a certain abstract goal—an ideal. The other question concerns the possible formulation of an innovation pathway that leads to a sufficient approximation of an ideal. Are engineers able to use the vision as determinative in their design decisions?

## Conclusion

This study adds to the growing body of literature regarding the role of visions, imaginaries and shared conceptions of 'the good life' in the development of sociotechnical systems. Ideals and utopian visions enable developments because they unite and mobilize actors around collective, and (potentially) unachievable, ends. Such ideals can guide thought and action on the levels of policy-making, engineering and public support.

However, this study also presents indications that, sometimes, idealized technological projects start to behave more and more like beacons themselves. They take over the navigational function of ideals. In some cases, this might not be problematic. In other cases, in which alternatives exist, and changes in society fail to inform new visions, idealized technologies and projects can become a hazard. They keep on demanding more research and more money. Technological unfeasibility and political ideals are here in conflict. This study argues that such projects may be hard to abandon, for that would mean abandonment of the ideal. The resulting stalemate, a back-and-forth between politicians, experts, and other stakeholders, can be obfuscating the potential of other projects.

Alternatively, idealistic aspirations to realize an unfeasible vision or set of ideals are not in vain. This study shows that ideals might lead to radically new perspectives on the design of energy systems. In these cases, ideals are productive, they impact the material sphere (Fig. 5) via innovation pathways in which ideals guide innovations for the long-term. Future research into the function and presence of ideals should therefore incorporate more sources to provide support for the arguments given in this study. This is especially true for the Severn Barrage case, in which the suggested interaction between (political) ideals and idealized projects needs to be more thoroughly addressed. Furthermore, this study recommends researching other energy projects and technologies in the current energy transition to renewables, and the way idealistic (political) discourse concerning these technologies/projects has developed.

The new perspective, developed in this study, is not only relevant for the analysis of inactivity regarding tidal power projects or developments in the design of nuclear technologies. It also provides more general insights into the guiding role of ideals for the development of energy systems. Once intertwined with specific projects, ideals may lead to conflicts, denialism, and empty promises. They fail to coordinate and motivate action, or lead to radical decisions that have no ground in (social) reality. In these cases, the tenacity of ideals becomes a liability.

## Data Availability

All data will be publicly available conforming with ERC-guidelines. 10.4121/14617575 (once published).

## Footnotes

1 See, for instance, Mazzucato’s *Mission Economy* (2021).

2 Rawls and Estlund focus on social issues only. In my opinion, this navigational function of ideals can be extended to innovation trajectories of technologies and the energy system at large. I develop my arguments for this position in the coming sections.

3 Whereas aesthetic ideals concern the perfect form.

4 This is an important issue in the contemporary debate about the use, if any, for Rawls' ideal justice. See, for instance, Sen (2009), Valentini (2012), Cohen (2009), Gaus (2016) and Estlund (2019), amongst others.

5 This can be seen as a second form of inability, namely, something that seems to be against the ‘nature’ of a social agent, or against the ‘deepest features of human psychology’ (Valentini, 2012, p. 660). Estlund (2011) calls this ‘limits to what humans will be able to muster the will to do’ (p. 207) despite their recognition of the desirability of the action.

6 Gaus, however, follows Sen (2009) in developing a strong criticism of the possibility of comparative judgment based on the ideal.

7 In line with previous and classic discussions of ideals as navigational tools, I stick here to the motif of the voyage towards utopia (Gaus, 2016).

8 When addressing safety as an engineering value, we ask the question: *How can we maximize a value given material, social, and epistemic constraints, and given possible conflicts with other values we seek to maximize?* This means that we expect value trade-offs and set value thresholds (van de Poel, 2009), such as minimum safety levels.

9 Evidently, utopia can be defined in many ways. Although often meaning ‘ideal society’, or ‘ideal community’, I here take it to refer to certain kind of visions, in which a blueprint is available for achieving a certain ideal in a certain society (Gaus, 2016; Rawls, 1971). In cases that this blueprint relies on a specific energy technology or project, we could call such a utopian vision a technical utopia, or energy utopia.

10 This projection proved highly exaggerated. At the beginning of the 1990s, approx. 420 reactors were in operation (Statista Research Department, 2022) compared to approx. 250 in 1980 (Char & Csik, 1987). The latest IAEA-report of May 2022, counts 439 operational reactors. This means the original, acceptable safety level of 1/10^4^ reactor years would entail, grosso modo, 1 serious reactor accident every 20 years.

11 ‘Inherent Safety refers to the achievement of safety through the elimination or exclusion of inherent hazards through the fundamental conceptual design choices made for the nuclear plant. Potential inherent hazards in a nuclear power plant include radioactive fission products and their associated decay heat, excess reactivity and its associated potential for power excursions, and energy releases due to high temperatures, high pressures and energetic chemical reactions. Elimination of all these hazards is required to make a nuclear power plant inherently safe. For practical power reactor sizes this appears to be impossible. Therefore the unqualified use of "inherently safe" should be avoided for an entire nuclear power plant or its reactor.’ (IAEA, 1991).

12 Mårtensson (1992) discusses four interpretations of inherent safety: ‘[1] One inherent risk factor would be eliminated; [2] At least one very important inherent risk factor would be eliminated; [3] All of the very important inherent risk factors would be eliminated; [4] All inherent safety hazards would be eliminated.’ (p.668) Only the last of these interpretations is an absolute, unfeasible principle that we could consider an ideal for reactor design.

13 ‘Note that we use the term inherently safer, not inherently safe, as we cannot avoid every hazard.’ (Kletz & Amyotte, 2019, p. 368).

14 Although I suggest Frenchness here as an ideal pursued in the design of nuclear technologies, what actually constituted this ideal, French identity, was something discovered and created through this design, rather than being a fixed goal from the beginning (Hecht & Callon, 2009). ‘Technology and Frenchness would shape each other’ (ibid, p. 52).

15 I cannot here further analyze the competing technopolitical regimes regarding atomic power that emerged in post-WW 2 France in the late 1950s. The Électricité de France, erected in 1946, focused on economic calculations, costs per Kwh, in their competitive reactor design. The ‘war of the systems’ (Hecht & Callon, 2009) ended in the late 1960s/beginning 1970s.

16 The archive at hansard.parliament.uk allows for a keyword search.

17 We can think, for instance, of standards and principles formulated in the Energy Justice literature, most notably by Sovacool and Dworkin (2015) in their *Energy Justice Decision-making Framework*: 'All people have a right to fairly access energy services', or 'All people should have access to high-quality information about energy and the environment, and fair, transparent and accountable forms of energy decision-making.' (p. 440).

18 Or, if thinking about not yet existing technologies, the kind of artefacts and systems we should be developing, the kind of R&D we should fund.
